# Fc fragment of IgG binding protein is correlated with immune infiltration levels in hepatocellular carcinoma

**DOI:** 10.17305/bb.2022.8586

**Published:** 2023-08-01

**Authors:** Yuhong Suo, Chunyu Hou, Guang Yang, Hongfeng Yuan, Lina Zhao, Yufei Wang, Ningning Zhang, Xiaodong Zhang, Wei Lu

**Affiliations:** 1 Department of Hepatobiliary Oncology, Liver Cancer Center, National Clinical Research Center for Cancer, Key Laboratory of Cancer Prevention and Therapy, Tianjin’s Clinical Research Center for Cancer, Tianjin Medical University Cancer Institute and Hospital, Tianjin Medical University, Tianjin, China; 2 Department of Gastrointestinal Cancer Biology, Tianjin Cancer Institute, Liver Cancer Center, National Clinical Research Center for Cancer, Tianjin Medical University Cancer Institute and Hospital, Tianjin, China

**Keywords:** Fc fragment of IgG binding protein (FCGBP), hepatocellular carcinoma (HCC), biomarker, Gene Set Enrichment Analysis (GSEA), immune infiltration

## Abstract

The Fc fragment of IgG binding protein (FCGBP) has been confirmed to play an important role in various cancers. However, the specific role of *FCGBP* in hepatocellular carcinoma (HCC) remains undefined. Thus, in this study, the enrichment analyses (Gene Ontology [GO], Kyoto Encyclopedia of Genes and Genomes [KEGG], and Gene Set Enrichment Analysis [GSEA]) of *FCGBP* in HCC and extensive bioinformatic analyses using data of clinicopathologic characteristics, genetic expression and alterations, and immune cell infiltration were perfomed. Quantitative real-time polymerase chain reaction (qRT-PCR) was used to verify the expression of *FCGBP* in both HCC tissues and cell lines. The subsequent results confirmed that *FCGBP* overexpression positively correlated with poor prognosis in patients with HCC. Additionally, *FCGBP* expression could effectively distinguish tumor tissues from normal tissues, which was verified by qRT-PCR. The result was further confirmed by using HCC cell lines. The time-dependent survival receiver operator characteristic curve exhibited the strong ability of *FCGBP* to predict survival in patients with HCC. Additionally, we revealed the strong relationship between *FCGBP* expression and a number of classic regulatory targets and classical oncogenic signaling pathways of tumors. Finally, *FCGBP* was involved in the regulation of immune infiltration in HCC. Therefore, *FCGBP* has potential value in the diagnosis, treatment, and prognosis of HCC and may be a potential biomarker or therapeutic target.

## Introduction

Primary liver cancer is one of the most common malignancies worldwide. Hepatocellular carcinoma (HCC) accounts for 75%–85% of the histologic forms of primary liver cancer, ranking third in cancer-related deaths [1]. Thus, an overwhelming burden has been imposed on public health and finance globally. Impressive progress has been made in HCC diagnosis and treatment [2], but most patients miss the optimal treatment opportunity at the time of diagnosis due to the characteristic of the occult onset of HCC [3]. Consequently, the clinical benefit and treatment options for patients are extremely limited. Generally, tumorigenesis is closely related to multiple factors, such as genetic alteration, tumor signal transduction pathways, tumor immune microenvironment, etc [4–7]. The roles of the immune system in anti-cancer strategies are vital [8]. The tumor microenvironment significantly influences the immunotherapy response and prognosis of patients [9]. Immunotherapies targeting tumor microenvironment are revolutionary in anti-cancer therapy [10]. Previous studies have shown that immunotherapy has achieved promising anti-tumor effects in many cancers, including HCC [8, 11–15]. Therefore, identifying the biomarkers closely related to the occurrence, development, and poor prognosis of HCC is crucial for early HCC diagnosis and proper treatment.

The Fc fragment of IgG binding protein (FCGBP) is expressed in many types of mucin-secreting cells and its secreted fluids, such as cells from the bronchus, colon, and cervix uteri [16]. FCGBP has been reported to be involved in maintaining the mucosal structure as a gel-like component of the mucosa [17]. Meanwhile, numerous studies revealed that *FCGBP* plays an important role in tumorigenesis and progression. *FCGBP* expression is different in various tumor types [18–20]. Cilibrasi et al. [21] revealed that *FCGBP* is highly expressed in glioblastoma. Wang et al. [22] confirmed that high *FCGBP* expression was associated with poor survival in ovarian cancer. In contrast, *FCGBP* was downregulated in gallbladder cancer [23]. Moreover, *FCGBP* expression levels were reported to positively correlate with the survival rate in colorectal cancer, including primary lesions and liver metastases [24]. However, the role of *FCGBP* in the prognosis and biological function of HCC is poorly understood.

This study analyzed the prognostic and diagnostic value of *FCGBP* in HCC. The correlation between *FCGBP* expression and the clinicopathological characteristics of patients was demonstrated. The potential pathogenic signaling pathways involving *FCGBP* were examined in HCC development. The correlation between *FCGBP* expression and immune infiltration was evaluated. Our finding provides insights into the mechanism by which *FCGBP* contributes to HCC.

## Materials and methods

### Analysis of prognosis and diagnosis

R software package “survminer” was used to construct the Kaplan–Meier (KM) survival curves. The Cancer Genome Atlas (TCGA) (https://portal.gdc.cancer.gov/), Tumor Immune Estimation Resource (TIMER) (https://cistrome.shinyapps.io/timer/), and Gene Expression Omnibus (GEO) databases (GSE14520) (https://www.ncbinlm.nih.gov/geo/) were used to evaluate the effect of *FCGBP* expression levels along with subgroups, including T stage, pathologic stage, and status of vascular invasion on HCC prognosis, as well as the effect of *FCGBP* expression levels on pan-cancer prognosis. Moreover, the receiver operator characteristic (ROC) curves, including diagnostic ROC curve and time-dependent survival ROC curve, nomogram model, and calibration plot, were constructed using R packages “pROC,” “ggplot2,” “timeROC,” “rms,” and “survival.”

### Expression analysis and genetic alteration analysis

The *FCGBP* gene expression data collected from the TCGA database included 50 patients with HCC with their paired adjacent normal liver tissues and 50 normal liver tissues with 374 HCC tissues. Meanwhile, the TIMER, GEO (GSE14520), Clinical Proteomic Tumor Analysis Consortium (http://ualcan.path.uab.edu/ analysis-prot.html), and Human Protein Atlas databases (https://www.proteinatlas.org/) were used to examine the differential *FCGBP* expression in pan-cancer and HCC. Additionally, the correlation between *FCGBP* expression levels and clinicopathological characteristics, including T stage, pathologic stage, and vascular invasion status, was computed using the R software package “ggplot2.”

Moreover, three datasets of HCC (INSERM, Nat Genet 2015; AMC, Hepatology 2014; TCGA, Firehose Legacy) collected from the cBioportal website (www.cbioportal.org/) were used to analyze the *FCGBP* genomic profiles. Meanwhile, the Catalogue of Somatic Mutations in Cancer (COSMIC) database (https://cancer.sanger.ac.uk/cosmic) was used to investigate the mutation types of *FCGBP* in HCC.

### Clinical tissue samples and cell lines

In 2020, 25 pairs of HCC and paratumor tissue samples were collected from Tianjin Medical University Cancer Institute and Hospital (Tianjin, China). These patients were pathologically diagnosed with HCC (without other malignancies) and had not received any special treatment preoperatively. The cell lines, LO2, HepG2, Huh7, and Hep3B, were purchased from ATCC (ATCC, Manassas, USA).

### RNA extraction and quantitative real-time polymerase chain reaction

The total RNA of tissues was extracted by TRIzol Reagent (ThermoFisher, CA, USA), following the manufacturer’s instructions. PrimeScript™ RT reagent kit (TaKaRa) was used to conduct the reverse transcription of RNA to cDNA. The SYBR Premix Ex Taq™ (TaKaRa) was used for quantitative real-time polymerase chain reaction (qRT-PCR). The primer sequences were as follows: *FCGBP*, 5′-GCCAAGGCTGAGATGATAGGC-3′(Forward) and 5′- CCTGCACAGAGATGGCAT AGT-3′ (Reverse); *GAPDH*, 5′-GGAGCGAGATCCCTC CAAAAT-3′ (Forward) and 5′-GGCTGTTGTCATACTTCTCATGG-3′ (Reverse). qRT-PCR parameters were: 95 ^∘^C for 5 min; (95 ^∘^C for 10 s, 55 ^∘^C for 20 s, and 72 ^∘^C for 30 s) × 35 amplification cycles. The relative *FCGBP* mRNA expression was normalized to *GAPDH* and analyzed using the 2^−ΔΔCT^ method.

### Difference and enrichment analysis

Data collected from the TCGA database for identifying the related differentially expressed genes according to the high-level and low-level groups of *FCGBP* were analyzed using the R software package “ggplot2.” Differences of |logFoldChange| of >1 and adjusted *P* value <0.05 were considered statistically significant. Then, we conducted GO term and Kyoto Encyclopedia of Genes and Genomes (KEGG) pathway enrichment analysis with R software packages “clusterProfiler,” “GOplot,” “ggplot2,” and “org.Hs.eg.db” on the collected differentially expressed genes to learn more about *FCGBP* information. Additionally, we further explored the potential biological signaling pathways by Gene Set Enrichment Analysis (GSEA), which collected the gene set of c2 (c2.cp.v7.2.symbols.gmt) from the Molecular Signatures Database (MSigDB). The adjusted *P* value < 0.05 and q-value (false discovery rate) < 0.25 indicated significant enrichment.

### Immune infiltration analysis

The TCGA database was utilized for analyzing the correlation of *FCGBP* expression levels with 24 types of immune cell infiltration in HCC using the R software package “GSVA.” Additionally, we evaluated the relationship between *FCGBP* expression and immune checkpoints using the TIMER and TCGA databases.

### Ethical statement

All specimens were anonymized in accordance with ethical and legal standards. This study was approved by the Ethics Committee of Tianjin Medical University Cancer Institute and Hospital (NO. E20210074) and all patients signed the written informed consents.

### Statistical analysis

This study used the R software V3.6.3 for data processing and statistical analysis. We used the Wilcoxon test for differential expression analysis. KM method, Cox regression, and log-rank test were used to evaluate the prognosis. Spearman’s correlation coefficients were calculated for correlation analysis. A *P* value < 0.05 was considered statistically significant.

**Figure 1.**
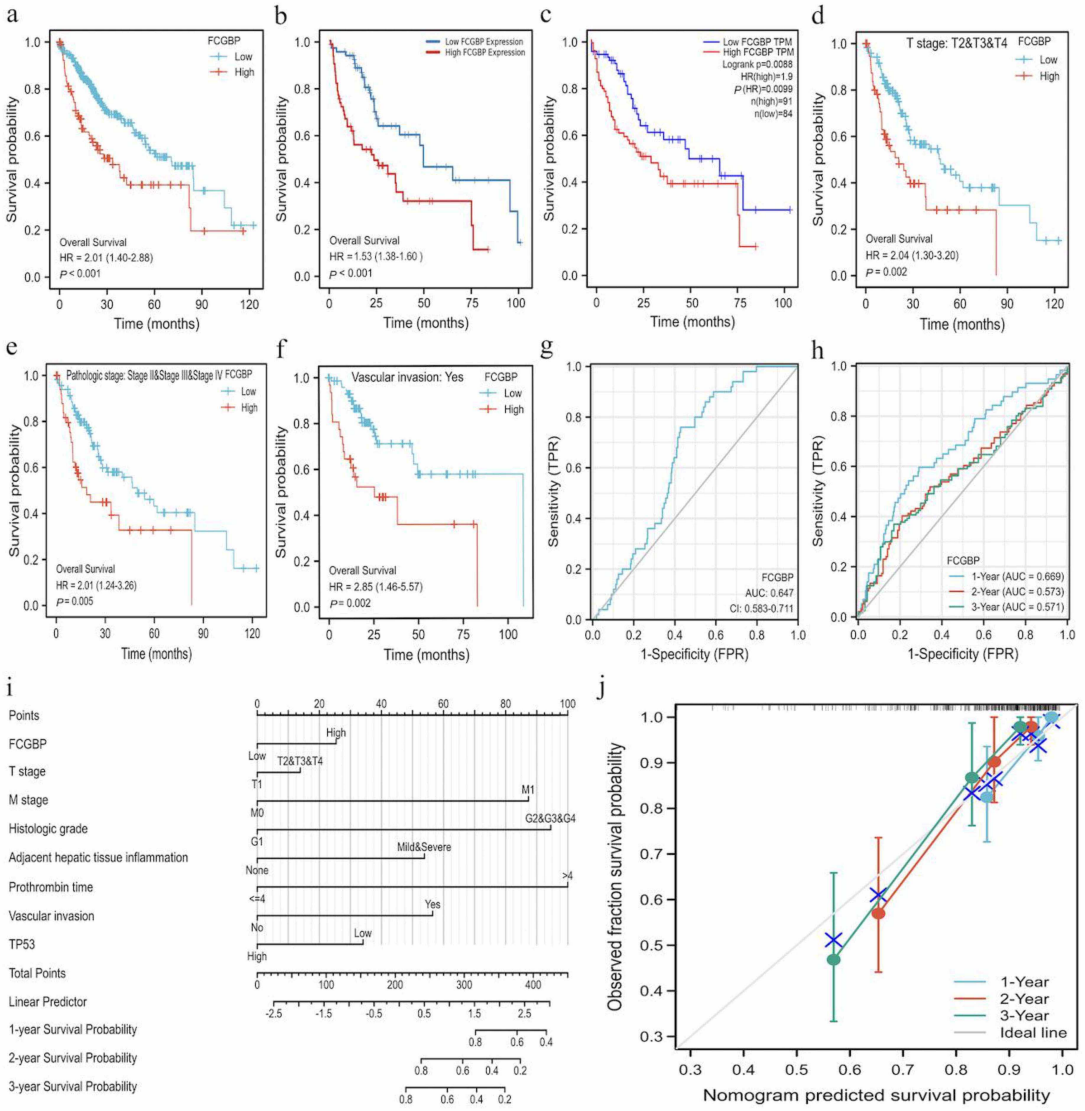
**Prognostic and diagnostic value of *FCGBP* in HCC.** (A-C) Relationship between *FCGBP* expression levels and OS in patients with HCC from (A) TCGA database, (B) TIMER database, and (C) Web-tool GEPIA. (D-F) Survival analysis for *FCGBP* expression stratified by (D) T2/T3/T4 stage, (E) stage II/III/IV, and (F) vascular invasion. (G) Diagnostic ROC curve to discriminate HCC tissues from normal tissues. (H) Time-dependent survival ROC curve to predict 1-, 2-, and 3-year OS for patients with HCC. (I) Nomogram model for predicting 1-, 2-, and 3-year OS. (J) Calibration curves were used to show the agreement between predicted probabilities by our nomogram model and actual probabilities. The closer the colored lines are to the gray line, the more accurate the nomogram model is. *FCGBP*: Fc fragment of IgG binding protein; HCC: Hepatocellular carcinoma; OS: Overall survival; TCGA: The Cancer Genome Atlas; TIMER: Tumor Immune Estimation Resource; ROC: Receiver operator characteristic; GEPIA: Gene Expression Profiling Interactive Analysis.

## Results

### Assessment of the prognostic significance and diagnostic value of *FCGBP* in HCC

A KM plot was constructed using the TCGA database to analyze the overall survival (OS), progression-free interval (PFI), and disease-specific survival (DSS) associated with *FCGBP* expression in HCC. We revealed that patients with higher *FCGBP* expression had shorter OS (hazard ratio [HR] ═ 2.01, *P* < 0.001) (Figure 1A), lower PFI (HR ═ 1.58, *P* ═ 0.005) (Figure S1a), and poorer DSS (HR ═ 1.68, *P* ═ 0.032) (Figure S1b). Concurrently, we confirmed the results by querying the TIMER, web-tool GEPIA, and GEO databases (Figures 1B and 1C and S2a). Then, we evaluated the relationships between *FCGBP* expression and OS of patients across 33 tumors (Figure S3). Our data revealed that *FCGBP* expression level could significantly affect the OS of breast invasive carcinoma (HR ═ 0.7, *P* ═ 0.033), colon adenocarcinoma (HR ═ 0.67, *P* ═ 0.05), head and neck squamous cell carcinoma (HR ═ 0.53, *P* < 0.001), kidney chromophobe carcinoma (HR ═ 6.24, *P* ═ 0.01), brain lower grade glioma (HR ═ 2.22, *P* < 0.001), liver hepatocellular carcinoma (LIHC) (HR ═ 2.03, *P* < 0.001), ovarian serous cystadenocarcinoma (HR ═ 1.88, *P* < 0.001), rectum adenocarcinoma (HR ═ 0.4, *P* ═ 0.021), sarcoma (HR ═ 1.54, *P* ═ 0.032), thyroid carcinoma (HR ═ 3.82, *P* ═ 0.008), uterine corpus endometrial carcinoma (HR ═ 0.63, *P* ═ 0.024), and uveal melanoma (HR ═ 0.24, *P* ═ 0.003) (Figure S4). Additionally, we explored the prognosis of patients with different clinicopathologic subgroups and *FCGBP* expression levels. The results revealed that patients with higher *FCGBP* expression, who were in T2/T3/T4 (HR ═ 2.04, *P* ═ 0.002), stage II/III/IV (HR ═ 2.01, *P* ═ 0.005), and vascular invasion (HR ═ 2.85, *P* ═ 0.002), were associated with poorer OS (Figure 1D–1F). Whereas the clinicopathological subtypes (including T1, pathologic stage I, and non-vascular invasion) were not statistically correlated with OS of patients with HCC (*P* > 0.05) (Figure S1c–S1e), suggesting the negative correlations between *FCGBP* expression levels and prognosis of patients with HCC.

The diagnostic ROC curve and time-dependent survival ROC curve were constructed to evaluate the accuracy of *FCGBP* expression levels in HCC diagnosis and prognosis prediction. The ROC analysis revealed that the different *FCGBP* expression levels could effectively distinguish the tumor tissues from the normal tissues (area under the curve [AUC]: 0.647) (Figure 1G). Moreover, the time-dependent survival ROC curve exhibited the strong discriminant ability of *FCGBP* to predict 1-year (AUC: 0.669), 2-year (AUC: 0.573), and 3-year (AUC: 0.571) OS for patients with HCC (Figure 1H), suggesting *FCGBP* as a potential diagnostic biomarker. We further constructed a nomogram model to predict the survival probabilities at 1, 2, and 3 years for patients with HCC, C-index: 0.794 (0.763–0.824) by integrating clinicopathologic factors, including *FCGBP* expression levels, T stage, M stage, TP53, adjacent hepatic tissue, inflammation, histologic grade, prothrombin time, and status of vascular invasion (Figure 1I). Additionally, a calibration plot was used to test the accuracy of the prediction model. Our data revealed that the predicted probabilities of our model fitted well with the actual probabilities (Figure 1J). Thus, we conclude that the prediction based on our nomogram model is reliable.

### Expression levels of *FCGBP* in pan-cancer and HCC

**Figure 2.**
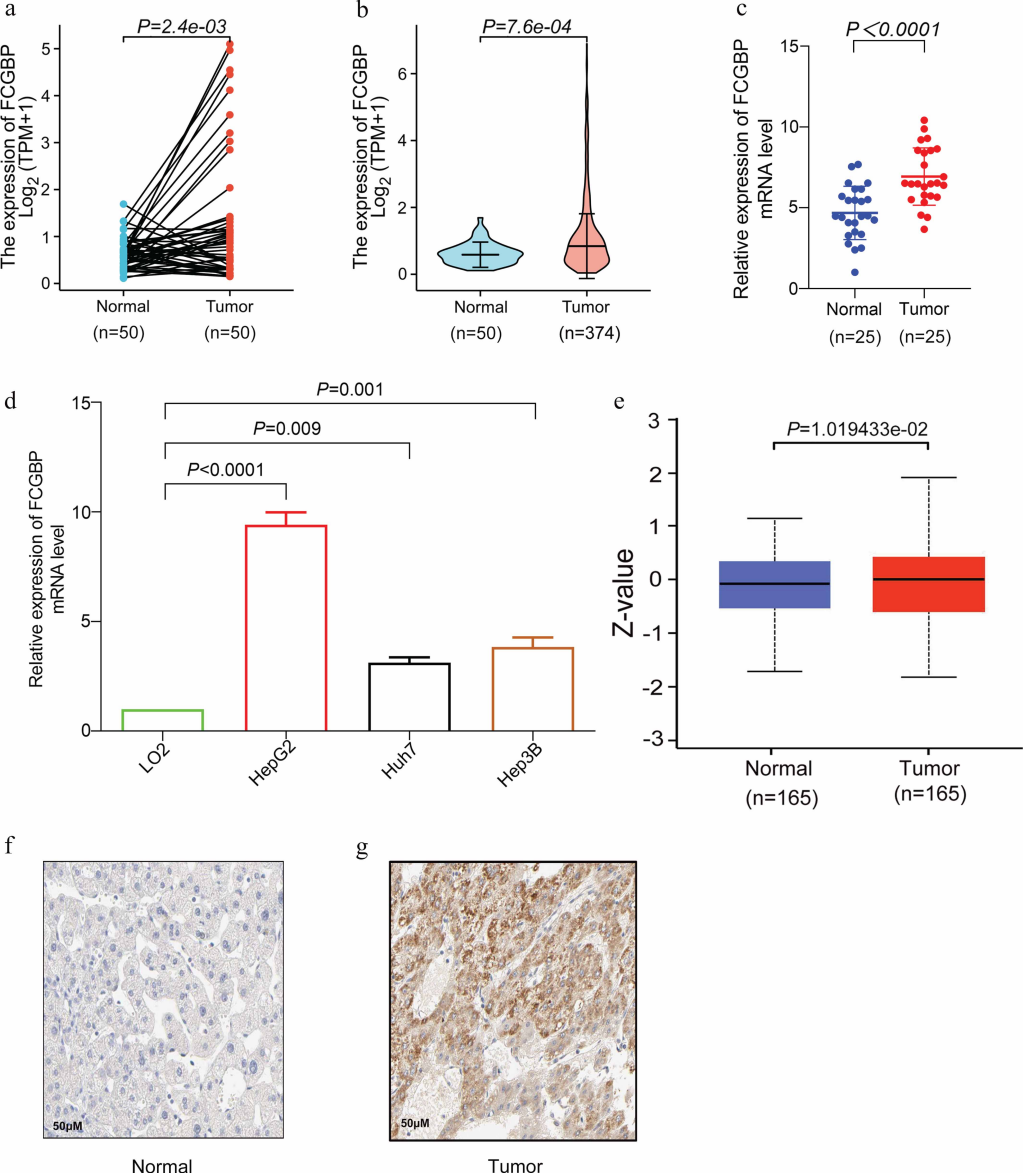
**The mRNA and protein expression levels of *FCGBP* in HCC.** (A) and (B) *FCGBP* mRNA expression levels in paired and unpaired HCC/normal samples from the TCGA database, respectively; (C) *FCGBP* mRNA expression levels were examined by qRT-PCR in 25 pairs of HCC and paratumor tissue samples; (D) The *FCGBP* mRNA level was evaluated in HCC and normal cell lines by qRT-PCR; (E–G) *FCGBP* protein expression levels in paired and unpaired HCC/normal samples, respectively. *FCGBP*: Fc fragment of IgG binding protein; HCC: Hepatocellular carcinoma; qRT-PCR: Quantitative real-time polymerase chain reaction; TCGA: The Cancer Genome Atlas.

We investigated *FCGBP* expression levels in various malignancies according to the paired samples and unpaired samples from the TCGA database to better understand the *FCGBP* functionality. The results revealed different *FCGBP* expression in various cancer types (Figure S5a and S5b). Interestingly, *FCGBP* was upregulated in various tumors (e.g., cholangiocarcinoma, glioblastoma multiforme, and HCC), but downregulated in some tumors, such as head and neck squamous cell carcinoma, kidney chromophobe carcinoma, kidney renal clear cell carcinoma, etc. Meanwhile, we analyzed the *FCGBP* expression levels in different cancer types using the data from the TIMER database, which were similar to the above data (Figure S5c). Moreover, we evaluated the *FCGBP* expression levels in HCC tissues. Our results demonstrated that *FCGBP* was highly expressed in HCC tissues at the mRNA and protein levels (Figures 2A, 2B, 2E–2G, and S2b). Meanwhile, qRT-PCR was used to validate this in 25 pairs of HCC and paratumor tissue samples (Figure 2C and Table S1) and HCC and normal cell lines (Figure 2D), suggesting that *FCGBP* overexpression may play an important role in HCC development.

### Analysis of *FCGBP* expression, clinicopathological parameters, and genetic alterations in HCC

**Figure 3.**
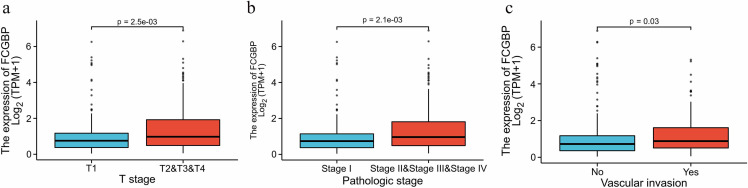
**Correlation between *FCGBP* mRNA expression levels and clinicopathologic characteristics in HCC.** (A) T stage; (B) Pathologic stage; (C) Status of vascular invasion. *FCGBP*: Fc fragment of IgG binding protein; HCC: Hepatocellular carcinoma.

The characteristics of 374 patients with HCC with complete RNAseq and clinical data collected from the TCGA database are shown in Table S2. Interestingly, we found that the *FCGBP* expression levels were significantly different in the different subgroups of T stage, pathologic stage, and status of vascular invasion (*P* < 0.05) (Figure 3A–3C). *FCGBP* expression levels were higher in patients with HCC with T2/T3/T4, stage II/III/IV, and vascular invasion than those in patients with HCC with T1 (*P* ═ 0.0025), stage I (*P* ═ 0.0021), and non-vascular invasion (*P* ═ 0.03). Additionally, we conducted a logistic regression analysis (Table 1) and found that *FCGBP* expression was closely associated with the T stage (odds ratio [OR] ═ 0.628 for T1 vs T2/T3/T4, *P* ═ 0.026), pathologic stage (OR ═ 0.589 for stage I vs stage II/III/IV, *P* ═ 0.014), and vascular invasion status (OR ═ 0.623 for no vs yes, *P* ═ 0.046), suggesting that *FCGBP* expression levels might be used to predict the HCC stage.

**Table 1:** Logistic regression analysis of *FCGBP* expression associated with clinicopathological parameters in HCC

**Characteristics**	**Total (*N*)**	**Odds Ratio (OR)**	***P* value**
T stage (T1 vs T2/T3/T4)	371	0.628 (0.416–0.945)	**0.026**
N stage (N0 vs N1)	258	1.000 (0.119–8.438)	1.000
M stage (M0 vs M1)	272	0.328 (0.016–2.601)	0.338
Pathologic stage (Stage I vs Stage II/III/IV)	350	0.589 (0.385–0.897)	**0.014**
Tumor status (Tumor free vs With tumor)	355	0.754 (0.494–1.148)	0.188
Sex (Female vs Male)	374	1.445 (0.936–2.240)	0.098
Race (Asian vs Black or African American and White)	362	1.051 (0.694–1.593)	0.813
Age (years) (≤60 vs >60)	373	0.832 (0.554–1.250)	0.377
Weight (kg) (≤70 vs >70)	346	1.359 (0.891–2.080)	0.156
Height (cm) (< 170 vs ≥170)	341	1.273 (0.827–1.965)	0.274
BMI (kg/m^2^) (≤25 vs >25)	337	1.143 (0.745–1.756)	0.539
Histologic grade (G1 vs G2/G3/G4)	369	0.674 (0.374–1.199)	0.183
Adjacent hepatic tissue inflammation (None vs Mild/Severe)	237	0.856 (0.512–1.429)	0.553
AFP (ng/mL) (≤400 vs >400)	280	0.708 (0.405–1.235)	0.224
Albumin (g/dL) (<3.5 vs ≥3.5)	300	0.597 (0.337–1.036)	0.070
Prothrombin time (s) (≤4 vs >4)	297	1.214 (0.735–2.019)	0.451
Child-Pugh grade (A vs B/C)	241	1.316 (0.541–3.415)	0.554
Fibrosis Ishak score (0 vs 1/2/3/4/5/6)	215	0.964 (0.545–1.696)	0.898
Vascular invasion (No vs Yes)	318	0.623 (0.390–0.991)	**0.046**

Values shown in bold are statistically significant (*P* < 0.05); *FCGBP*: Fc fragment of IgG binding protein; HCC: Hepatocellular carcinoma; BMI: Body mass index; AFP: Alpha fetoprotein.

Next, we evaluated the *FCGBP* genetic alterations in HCC using three databases (TCGA, Firehose Legac; AMC Hepatology 2014; and INSERM, Nat genet 2015). Three types of genetic alterations were found (missense mutation, truncating mutation, and amplification), with a 4% rate of *FCGBP* genetic alteration (33/851 patients), and the missense mutation was the most prevalent type of genetic alteration (Figure S6a). The mutation frequency of *FCGBP* was 3% (26/851 patients) (Figure S6b). The rate of *FCGBP* genetic alteration ranked from 3.29% (8/243 patients) to 4.51% (17/377 patients) and the mutation frequency ranged from 2.6% (6/231 patients) to 3.29% (8/243 patients) (Figure S6b). We performed an analysis using the COSMIC database to gain further insight into the *FCGBP* mutations. The missense substitution remained the most prevalent type of *FCGBP* mutations (63.47%, 384/605 samples) (Figure S6c). C>T was the most prevalent type of substitution mutation (40.07%, 216/539 samples) (Figure S6d). Additionally, we assessed the effect of the *FCGBP* genetic alterations on patients’ survival using KM plots and log-rank tests and found little effect of this alteration on OS and disease-free survival (Figure S6e and S6f).

### Differential expression analysis associated with *FCGBP* and enrichment analysis

**Figure 4.**
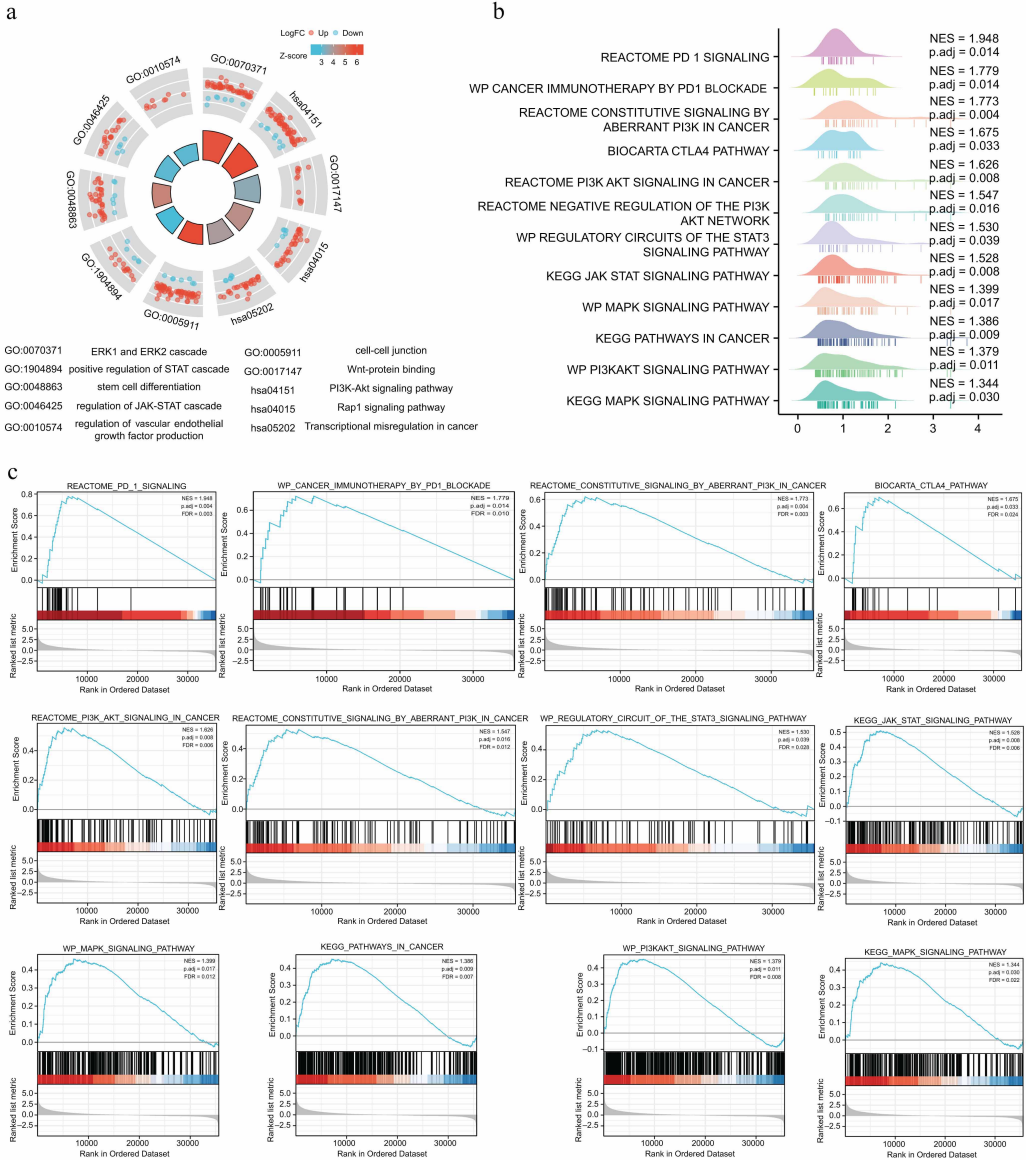
**Enrichment analysis of differentially expressed genes related to *FCGBP* expression in HCC.** (A) GO and KEGG enrichment analysis; the height of bars located in the center of the circle represents the adjusted *P* value and the higher the bars, the smaller the adjusted *P* value; the color of bars represents the Z-score value, and the higher the Z-score value, the stronger the regulation; (B) Visualization of GSEA; (C) GSEA. *FCGBP*: Fc fragment of IgG binding protein; HCC: Hepatocellular carcinoma; GO: Gene Ontology; KEGG: Kyoto Encyclopedia of Genes and Genomes; GSEA: Gene Set Enrichment Analysis; NES: Normalized enrichment scores; p.adj: Adjusted *P* value.

A total of 2219 differentially expressed genes were identified by single gene differential expression analysis of high- and low-expression *FCGBP* samples using the TCGA database (Figure S7). Next, we performed the GO and KEGG enrichment analysis, including GO:0010574, GO:0046425, GO:0048863, GO:1904894, GO:0005911, GO:0070371, GO:0017147, hsa04151, hsa05202, and hsa04015 (Figure 4A and Table S3), suggesting that *FCGBP* expression is strongly related to some classic regulatory targets of tumors and classical oncogenic signaling pathways, such as extracellular regulated protein kinases (ERK), signal transducer and activator of transcription (STAT), phosphatidylinositol-3 kinase (PI3K)/AKT signaling pathway, etc. GSEA based on the single gene differential expression analysis was conducted to further explore the differentially activated signaling pathways associated with *FCGBP* expression levels. The finding revealed that the signaling pathways related to immune checkpoints, PI3K/AKT, Janus kinase (JAK)/STAT, mitogen-activated protein kinase (MAPK), and cancer were significantly enriched in the group of high *FCGBP* expression (Figure 4B and 4C), suggesting the oncogenic role of *FCGBP* by regulating multiple targets and activating multiple signaling pathways in HCC.

### Correlation between *FCGBP* expression levels and immune infiltration in HCC

**Figure 5.**
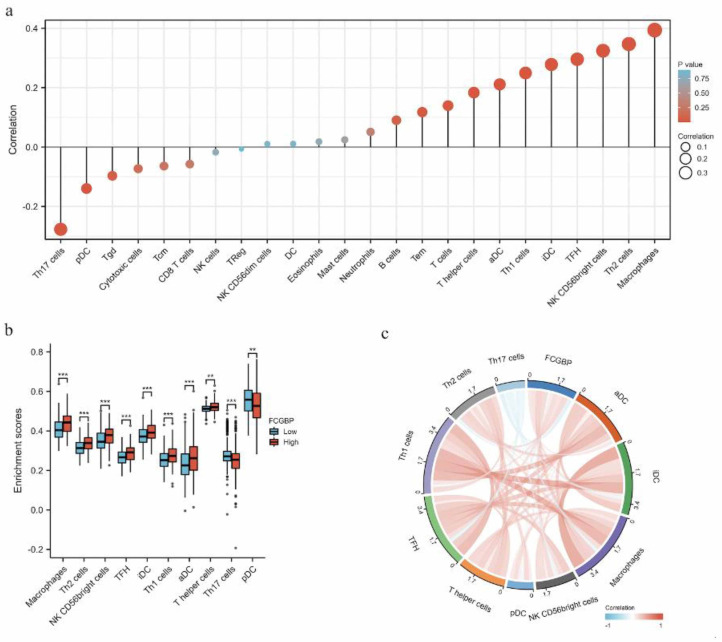
**Association between *FCGBP* expression and immune cells in HCC.** (A) Relationships between *FCGBP* expression levels and 24 types of immune cells in HCC; (B) The immune cells of striking differences between the high and low groups of *FCGBP* expression; (C) Visualization of the correlation between *FCGBP* expression and immune cells; red represents a positive correlation, blue represents a negative correlation, and deeper the color, the stronger the correlation. *FCGBP*: Fc fragment of IgG binding protein; HCC: Hepatocellular carcinoma; NK: Natural killer; TFH: T follicular helper; iDC: Immature dendritic cells; aDC: Activated dendritic cells; pDC: Plasmacytoid dendritic cells.

**Figure 6.**
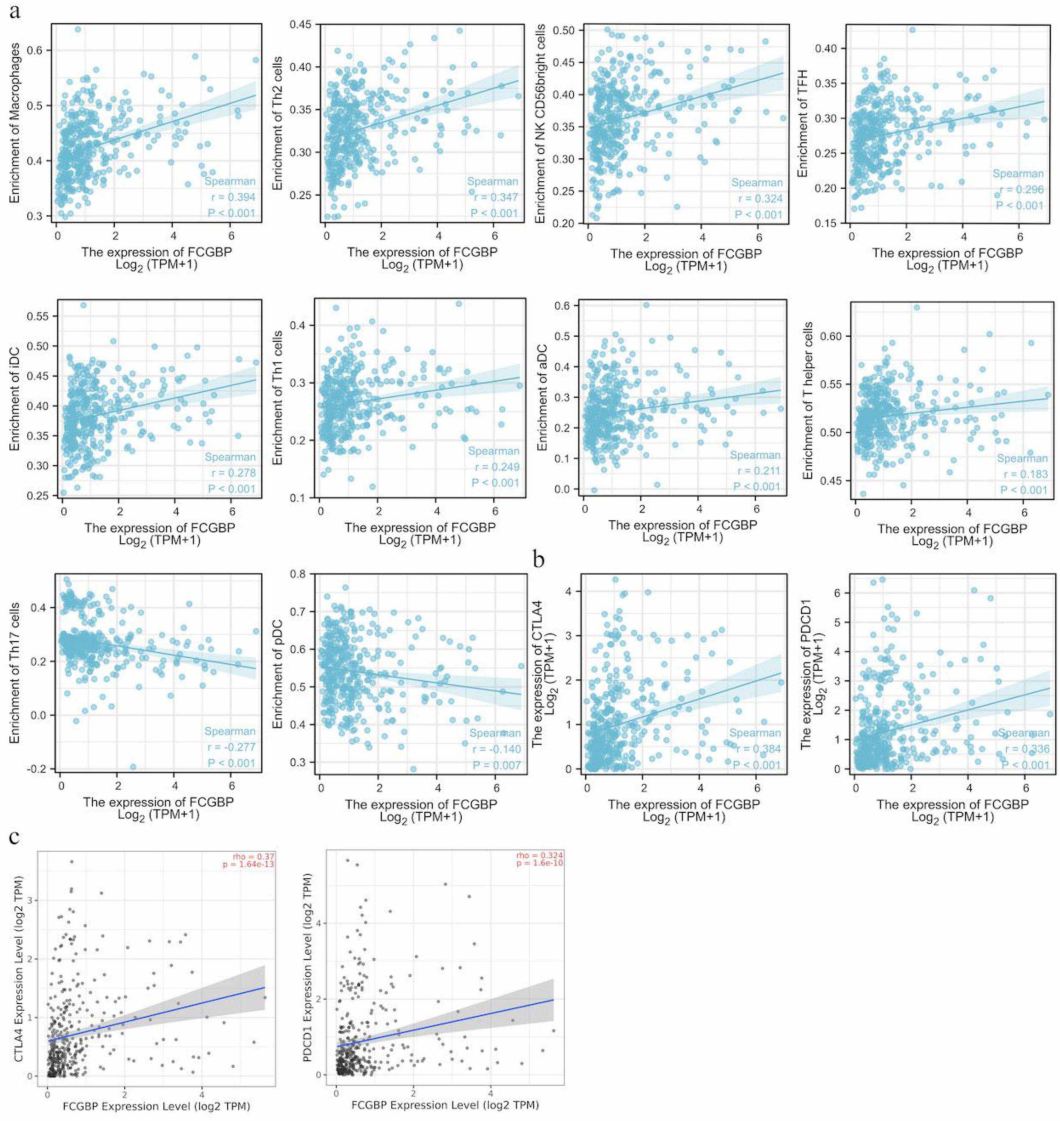
**Correlation between *FCGBP* expression and the top 10 immune cells of differences or immune checkpoints.** (A) The correlation of *FCGBP* expression with immune infiltration level of macrophages, Th2 cells, NK CD56 bright cells, TFH, iDC, Th1 cells, aDC, T helper cells, Th17 cells, and pDC; (B) The correlation of *FCGBP* expression with CTLA-4 and PDCD1 based on the TCGA database; (C) The correlation of *FCGBP* expression with CTLA-4 and PDCD1 based on the TIMER database. *FCGBP*: Fc fragment of IgG binding protein; HCC: Hepatocellular carcinoma; TCGA: The Cancer Genome Atlas; TIMER: Tumor Immune Estimation Resource; NK: Natural killer; TFH: T follicular helper; iDC: Immature dendritic cells; aDC: Activated dendritic cells; pDC: Plasmacytoid dendritic cells; CTLA-4: Cytotoxic T-lymphocyte-associated protein 4; PDCD1: Programmed cell death protein 1.

Generally, the occurrence and development of malignant tumors and immunity are closely interlinked [25]. Therefore, we analyzed the relationship between *FCGBP* expression levels and 24 types of immune cells in HCC (Figure 5A). Figures 5B, 5C, and 6A show that the *FCGBP* expression was positively associated with some immune cells, such as macrophages (*r* ═ 0.394, *P* < 0.001), T helper 2 cells (*r* ═ 0.347, *P* < 0.001), natural killer CD56 bright cells (*r* ═ 0.324, *P* < 0.001), T follicular helper cells (*r* ═ 0.296, *P* < 0.001), immature dendritic cells (*r* ═ 0.278, *P* < 0.001), T helper 1 cells (*r* ═ 0.249, *P* < 0.001), activated dendritic cells (*r* ═ 0.211, *P* < 0.001), and T helper cells (*r* ═ 0.183, *P* < 0.001). However, T helper 17 (Th17) cells (*r* ═ −0.277, *P* < 0.001) and plasmacytoid dendritic cells (*r* ═ −0.14, *P* ═ 0.007) were negatively correlated with *FCGBP* expression levels. Additionally, the relationship between the *FCGBP* and immune checkpoints expression levels was analyzed using TCGA and TIMER databases (Figure 6B and 6C). We found that the *FCGBP* expression was positively correlated with that of cytotoxic T-lymphocyte-associated protein 4 (CTLA-4) and programmed cell death protein 1 (PDCD1) (*P* < 0.001), suggesting that *FCGBP* might play an important role in HCC occurrence and development by regulating immune infiltration.

## Discussion

HCC remains a global public health challenge due to the low rates of early diagnosis and high mortality and incidence rates. The low rates of early diagnosis are one of the major causes of death in patients with HCC [26]. Importantly, its mortality and incidence rates are increasing [27]. By 2025, more than 1 million people are expected to suffer from liver cancer each year, which will put a huge economic burden on society, especially in East Asia and Africa [27, 28]. Thus, improving early detection rates and exploring new effective treatments for patients with HCC is urgently needed. In recent years, we can study more fundamental molecular mechanisms of HCC with the rapid advancement of precision medicine and sequencing technologies. Many studies have confirmed that *FCGBP* plays an important role in various malignancies, including ovarian cancer [22, 29], glioblastoma [21], colorectal cancer [20, 24, 30, 31], gallbladder cancer [23], head and neck squamous cell carcinoma [32, 33], prostate cancer [34], papillary thyroid carcinoma [35], and osteosarcoma [36]. However, to date, the role of *FCGBP* in HCC has not been reported. Herein, we conducted a comprehensive analysis of *FCGBP* in HCC using multiple public databases, clinical tissue samples, and cell lines.

This study revealed that the higher the *FCGBP* expression levels, the worse the prognosis of patients with HCC. Meanwhile, ROC curve analysis and the nomogram based on *FCGBP* expression revealed that *FCGBP* has certain values in diagnosing and determining the prognosis of patients with HCC. Additionally, aberrant *FCGBP* expression was found in most human cancers, including liver cancer. We confirmed significantly upregulated *FCGBP* expressions in HCC samples at mRNA and protein levels compared with normal samples. *FCGBP* expression was positively associated with clinicopathological characteristics, including T stage, pathologic stage, and vascular invasion status. Therefore, *FCGBP* may be a potential prognostic and diagnostic biomarker for patients with HCC.

Next, we studied *FCGBP* mutations in patients with HCC. Our finding revealed that the mutation frequency of *FCGBP* was 3%, with the predominant type being a missense mutation. However, no significant correlation was found between these mutations and prognosis, which may be due to the small sample size. Hence, we will further expand the sample size to explore its clinical significance.

We performed GO, KEGG, and GSEA enrichment analyses to further elucidate the underlying molecular mechanisms of *FCGBP* in HCC. GO analysis indicated that *FCGBP* was involved in the tumor initiation and progression through various pathways, including ERK1 and ERK2 cascade, JAK-STAT cascade regulation, vascular endothelial growth factor production regulation, etc. These ways played a crucial role in HCC occurrence and development [37–39]. KEGG analysis revealed that *FCGBP* was related to multiple oncogenic signaling pathways, especially PI3K/AKT signaling pathway. Several studies have confirmed the cancer-promoting effects of these signaling pathways in HCC [40–43]. Additionally, we performed GSEA to further explore the oncogenic signaling pathways of *FCGBP*. We found that the multiple typical carcinogenic pathways and immune escape-related pathways were significantly enriched, such as PI3K/AKT, MAPK, JAK/STAT, and CTLA-4 and PDCD1 signaling pathways, in which *FCGBP* expression was increased. The main results of these enrichment analyses were in accordance. Thus, we conclude that *FCGBP* plays an important role in HCC occurrence and development by regulating multiple tumorigenic targets and activating multiple carcinogenic signaling pathways.

Studies revealed that immune cells played an important role in regulating the malignant behaviors of tumor cells [44–46]. Therefore, we performed an analysis for immune infiltration, which indicated that *FCGBP* expression levels were closely related to multiple immune cells in HCC, which was consistent with the results of the previous report [47]. Additionally, immune checkpoint inhibitors provided a new direction for treating malignancies [48]. Our study revealed that *FCGBP* expression was closely associated with CTLA-4 and PDCD1. It suggests that *FCGBP* is a potentially novel therapeutic target in HCC.

Our study comprehensively evaluates the potential value of *FCGBP* and explores its molecular mechanisms in HCC occurrence and progression using the cross-validation method across multiple databases and qRT-PCR; however, the above findings need to be further verified in the wet laboratory.

## Conclusion

Taken together, our study preliminarily identified the role and mechanisms of *FCGBP* in HCC occurrence and development. Our finding provides insights into the role of a potential HCC biomarker in terms of diagnosis, prognosis, and treatment.

## Supplemental Data

Supplementary data are available at the following link: https://www.bjbms.org/ojs/index.php/bjbms/article/view/8586/2711.
